# A Study on the Radiation Resistance Performance of an Al_2_O_3_ Composite Tritium Permeation Barrier and Zirconium-Based Tritium-Absorbing Materials

**DOI:** 10.3390/ma17225600

**Published:** 2024-11-15

**Authors:** Changzheng Li, Rui Shu, Yinghong Li, Long Wang, Runjie Fang, Lihong Nie, Qisen Ren, Xiang Liu, Jing Hu, Shaohong Zhang

**Affiliations:** 1 School of Physics, Zhejiang University, Hangzhou 310058, China; lichangzheng@cgnpc.com.cn (C.L.); zhangshh@zju.edu.cn (S.Z.); 2 China Nuclear Power Technology Research Institute, Shenzhen 518031, China; shurui@cgnpc.com.cn (R.S.); liyinghong@cgnpc.com.cn (Y.L.); fangrunjie@hotmail.com (R.F.); nielihong@cgnpc.com.cn (L.N.); renqisen@cgnpc.com.cn (Q.R.); 3 Southwest Institute of Physics, Chengdu 610225, China; wanglong@swip.ac.cn

**Keywords:** tritium permeation barrier (TPB), irradiation, permeation reduction factor (PRF)

## Abstract

The permeation of tritium from secondary neutron source rods in nuclear power plants presents a significant and unavoidable safety concern both for internal equipment and the external environment. This study primarily explores two feasible strategies for tritium permeation barriers: coating stainless steel surfaces with tritium permeation barrier (TPB) materials and utilizing materials with excellent tritium absorption properties. Through external ion irradiation tests, a comparative analysis was conducted on the tritium permeation performance, morphology, and nanohardness changes in two tritium-resistant designs, specifically Cr_2_O_3_/Al_2_O_3_ composite coatings and a zirconium-based tritium-absorbing material under varying irradiation doses. The results indicate that both approaches exhibit exceptional radiation resistance, maintaining an effective tritium permeation reduction factor (PRF) even after irradiation.

## 1. Introduction

In pressurized water reactor (PWR) nuclear power plants, the secondary neutron source (Sb-Be) produces tritium after irradiation [1]. Tritium is radioactive and highly permeable, posing significant hazards to operators and the environment by contaminating reactor coolant and leading to hydrogen embrittlement of structural materials. Controlling tritium permeation and leakage is an essential issue in evaluating the radiation environment in PWRs.

Tritium formation in secondary neutron source rods mainly occurs through three reactions, with the primary reaction occurring during reactor operation, as shown in Equations (1)–(3). For a 900 MW power plant [2] with a neutron flux of 3.5 × 10^14^ n·cm^−2^·s^−1^ and neutron energy E ≥ 0.625 eV, each gram of Be in the core can produce approximately 2.47 × 10^12^ atoms/s, including 2.30 × 10^12^ helium atoms and 1.7 × 10^11^ tritium atoms. Assuming all of the tritium remains in the rod, at the end of the secondary neutron source component’s lifetime, the partial pressures of helium and tritium inside the rod could reach around 7 MPa and 0.5 MPa, respectively. However, in reality, the Sb-Be core cannot retain tritium, and the stainless-steel cladding currently used enables high tritium permeability, leading to nearly all of the tritium produced by the Sb-Be core entering the coolant [3]. In French 1300 MW and 1450 MW nuclear power plants, tritium from the secondary neutron source components accounts for about 20–40% of the tritium in the primary coolant system [4]. Around the year 2000, the tritium produced by secondary neutron sources at Daya Bay accounted for approximately 10–20% of the annual emissions. Abnormal axial power shifts (AOAs) in the core can also increase tritium production in secondary neutron source rods, as the axial power peak shifts towards the bottom, where the secondary neutron sources are located, resulting in increased tritium generation proportional to the neutron flux in the core. Implementing effective measures to trap tritium within secondary neutron source rods is a viable strategy to reduce tritium release.

(1) B49e+n01→α24+H26eH26e→t=807msβ−−10+L36iL36i+n01→α24+H13

(2) B49e+n01→B410e+γB410e+n01→L38i+H13

(3) B49e+n01→L37i+H13L37i+n01→H25e+H13

Tritium permeates by diffusion in the form of interstitial atoms and is able to pass through almost all metals with high permeability [5]. Tritium permeability increases with increasing temperature and neutron flux. The permeability of hydrogen isotopes (hydrogen, deuterium, and tritium) in stainless steel at temperatures greater than 400 °C is two to three orders of magnitude higher than at room temperature. In addition, the permeability of stainless steel under irradiation in the reactor will be higher than that measured under the same temperature and pressure conditions in off-heap tests. For example, experimental results for 304 stainless steel indicate that, at 570 °C, the permeability under irradiation conditions is three times higher than that under non-irradiation conditions [6]. The slow growth of the oxide layer on the surface of stainless steel will lead to a decrease in permeability. For example, under irradiation conditions, a 0.01 vol.% oxide layer on 0.12C-18Cr-10Ni-Ti can reduce its permeability by around 100 times [7]. The permeability of 316 stainless steel inside the stack is approximately two to five times higher than the permeability measured in external tests [8].

Coating structural materials with a TPB layer of sufficient thickness with a low tritium diffusion coefficient and a low surface recombination constant is one of the most effective practical methods to reduce tritium permeation while preserving the overall properties of the structural materials. The tritium permeation capability of a coating is generally evaluated using the PRF (tritium permeation reduction factor), defined as the ratio of tritium permeability in the substrate material to that in the coated substrate. A higher PRF indicates stronger tritium permeation resistance. Al_2_O_3_ coatings are considered important candidates for tritium permeation barriers due to their high PRF (up to 2–4 orders of magnitude) [9], ease of preparation, and corrosion resistance. However, differences in thermal expansion coefficients between the substrate and coating materials can cause aluminum oxide coatings to peel off, posing a significant technical challenge. This can be addressed using composite coatings (e.g., Al_2_O_3_/FeAl, Al_2_O_3_/TiC, Al_2_O_3_/Cr_2_O_3_, Al_2_O_3_/SiC, and Al_2_O_3_/TiB_2_), which improve adhesion to the substrate, provide effective stress relief, and exhibit self-healing capabilities for microcracks. These enhancements increase the coating’s resistance to spalling and introduce tritium diffusion traps, thereby improving tritium permeation resistance [10].

Aluminide coatings have been widely used as anti-corrosion coatings in conventional industries and the preparation techniques are well established. K. Stein [11] used the liquid hot-dip aluminum coating technique to prepare aluminde coatings on MANETs, obtaining hydrogen PRFs of 260–1000 at 300–470 °C. Benamatic [12] prepared aluminized coatings on MANET using a solid aluminizing technique and obtained a hydrogen PRF of 2500~5000 °C at 400~500 °C. Fazio [13] prepared aluminide coatings on MANET surfaces using spraying, low-pressure plasma sputtering, and air plasma sputtering, respectively, with hydrogen PRFs of 1–3 orders of magnitude in the range of 350–500 °C. A. Perujo [14] prepared aluminide coatings on MAET II using vacuum plasma sputtering with hydrogen PRFs of 2–3 orders of magnitude at 300~550 °C. Forcey [12] prepared aluminide coatings on 316L and DIN 1.4914 by solid aluminizing and obtained hydrogen PRFs of 2–4 orders of magnitude at 300~500 °C. Liu Xingzhao [15] obtained PRFs of 300~400 after TiN plating on HR-1 by CVD. Forcey [9] prepared TiN/TiC and Al_2_O_3_/TiN/TiC composite coatings on a 316L surface using the CVD method and found that the PRF of deuterium is only one order of magnitude at 250~450 °C. While Shang Changqi [16] found the PRFs of TiC monolayer film and TiN+TiC composite film prepared on a 316L surface using the CVD method to be 5~6 orders of magnitude at 200~600 °C. Yao Zhenyu [17] prepared TiN+TiC+TiN and TiN+TiC+SiO_2_ composite films on 316L stainless steel, which have a PRF of 4~5 and 4~6 orders of magnitude, respectively, at 200~600 °C, using the physical vapor deposition (PVD) method.

Zirconium-based alloys are also widely used in hydrogen storage and purification systems due to their strong affinity for tritium. Tritium formed from fuel fission is nearly entirely sealed in the fuel cladding as hydride, with only about 0.01% of tritium eventually leaking from nuclear power plants [4]. Studies by Song Jiangfeng et al. [18] showed that Zr_2_Fe alloy powder has a hydrogen absorption capacity of about 32% under 300 °C and 1 bar conditions with a 2.5% H_2_/Ar mixture. In 2000, the US Department of Energy designed a Tritium Producing Burnable Absorber Rod (TPBAR) using zirconium alloys as tritium-absorbing materials in 17 × 17 fuel assemblies [19]. However, mature studies on the use of treated zirconium-based alloys as secondary neutron source tritium barriers in PWR environments are lacking. The impact of irradiation dose on the tritium permeation performance of coatings or zirconium-based alloys, given the long service time of secondary source rods in reactors, is crucial for their application.

This study evaluates and selects tritium permeation barriers with excellent performance through a series of tests on Cr_2_O_3_/Al_2_O_3_ coatings and zirconium alloy self-oxidation films. It examines the tritium permeation performance of 316L stainless steel, Cr_2_O_3_/Al_2_O_3_ coatings, and zirconium alloy self-oxidation films before and after irradiation. The influence of factors such as irradiation, temperature, and deuterium partial pressure on the tritium permeation performance of the coatings and oxidation films is assessed. Based on experimental results, the tritium permeation behavior of coated stainless steel tubes and pre-oxidized zirconium alloy tubes is simulated, identifying key factors affecting the materials’ tritium permeation resistance.

## 2. Experimental Methods

### 2.1. Irradiation Tests

**Figure 1:**
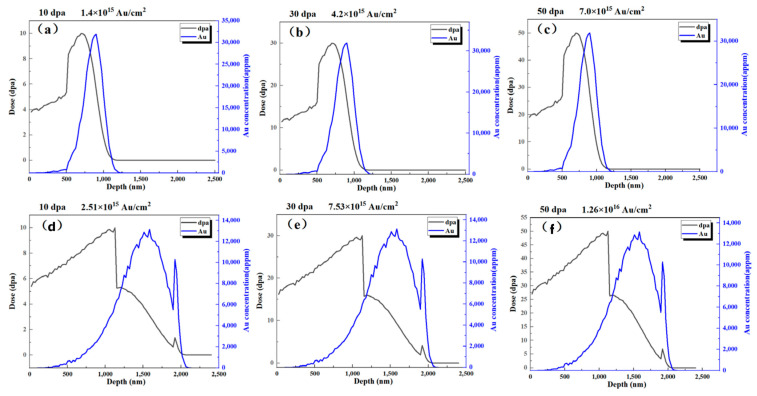
The distribution of displacement damage and Au ion concentration: (**a**–**c**) Cr_2_O_3_/Al_2_O_3_ coatings in different irradiation doses, (**d**–**f**) pre-oxidized zirconium alloy in different irradiation doses.

The tests primarily used an HVE 3MV tandem accelerator. Gold ions were chosen for irradiation because they are chemically inert and do not react with the material. Additionally, the concentration distribution and damage region of gold ions differ slightly near the surface, causing minimal impact on material composition. Using ions like Fe or Al, which are present in the material, for irradiation would interfere with microstructure and composition analysis and would have a more significant impact on the material’s phase structure, differing from actual neutron irradiation conditions, thereby not effectively simulating or evaluating the impact of neutron irradiation damage on the coatings; the test parameters are shown in Table 1. Figure 1 shows the distribution of displacement damage and Au ion concentration as a function of depth for different irradiation doses (10 dpa, 30 dpa, and 50 dpa).

For the Al_2_O_3_ coating on 316L stainless steel, Au ion irradiation doses of 1.4 × 10^15^ Au/cm^2^ (10 dpa), 4.2 × 10^15^ Au/cm^2^ (30 dpa), and 7.0 × 10^15^ Au/cm^2^ (50 dpa) were applied, covering an irradiated depth extending to approximately 1.5 μm from the surface. For the ZrO_2_ coating on 316L stainless steel, Au ion irradiation doses of 2.51 × 10^15^ Au/cm^2^ (10 dpa), 7.53 × 10^15^ Au/cm^2^ (30 dpa), and 1.26 × 10^15^ Au/cm^2^ (50 dpa) were used, with the irradiated region extending to a depth of approximately 1.9 μm.

### 2.2. Hydrogen Gas Isotope Permeation (GDP) Test

**Figure 2:**
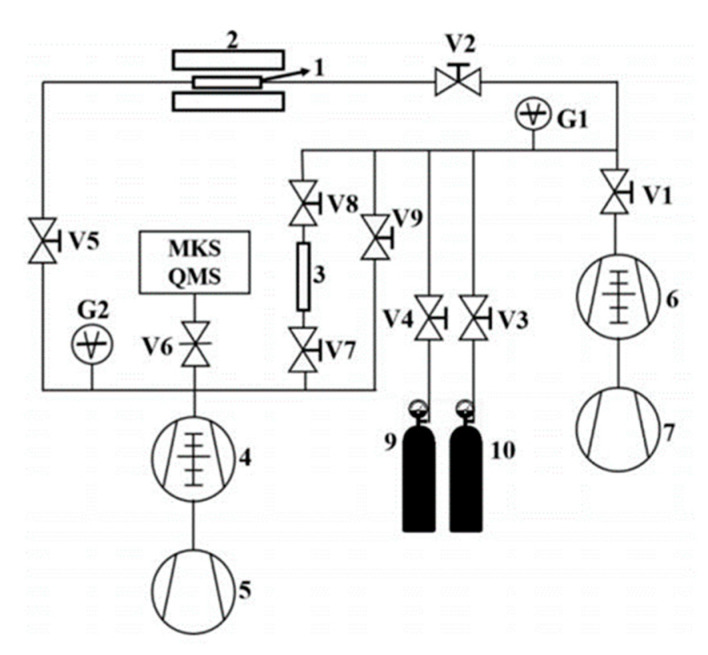
Schematic diagram of deuterium permeation testing system: 1—sample, 2—furnace, 3—standard leak, 4—ultra-high vacuum system, 5—mechanical pump, 6—high vacuum system, 7—mechanical pump, 9~10—gas cylinder, V1~9—valves.

Deuterium is used for permeation experiments. The structure of the experimental device used in this experiment is shown in Figure 2.

The deuterium permeation test conditions for the coatings after irradiation are shown in Table 2. At least one sample of each coating type is prepared for the permeation test under each set of irradiation parameters.

### 2.3. Microstructure Inspection

The microstructural and compositional analyses of the specimens before and after irradiation were conducted using a combination of advanced imaging techniques. Focused ion beam (FIB) milling with the FEI Helios NanoLab 600i was employed to prepare the samples, achieving a typical thickness of around 5 μm to meet the criteria required for transmission electron microscopy (TEM) examination. The TEM analysis, performed with the FEI Talos F200X and FEI Tecnai G2 F20, provided the high-resolution imaging essential for examining irradiation-induced microstructural features, such as dislocations, phase distributions, and grain boundary orientations. Additionally, selected area electron diffraction (SAED) was used to determine crystallographic orientations, enhancing our understanding of structural changes under irradiation.

For surface and cross-sectional morphological and compositional characterization, a field emission scanning electron microscope (FESEM), specifically the Thermo Scientific Apreo 2c, was used. While TEM offered high-resolution insights into microstructure, the FESEM provided broader topographical details of both surfaces and cross-sections. Combined with energy-dispersive X-ray spectroscopy (EDS), the FESEM enabled detailed elemental mapping and compositional analysis across the irradiated samples, revealing the uniformity and distribution of key elements. This combination of SEM and EDS provided a complementary view to the TEM data, particularly for assessing the compositional stability and morphological integrity of the irradiated coatings.

### 2.4. Nanoindentation Inspection

Nanoindentation tests were conducted on the samples before and after irradiation to assess the impact of irradiation on the mechanical properties of the coatings, specifically focusing on adhesion performance. The Nano Indenter G200 (Agilent Technologies Inc., Santa Clara, CA, USA) with a Berkovich diamond tip was used, featuring a tip radius of approximately 100 nm. The thickness of the Cr_2_O_3_/Al_2_O_3_ coatings was about 1.5 μm and the indentation depth, which was controlled within 5% of the coating thickness to minimize substrate influence, was approximately 75 nm.

The nanoindentation experiments employed two modes: continuous stiffness measurement (CSM) and the static mode. The calculation of nanohardness was based on the Oliver–Pharr method, which allows for the determination of hardness and elastic modulus from the load–displacement data collected during the indentation process. To ensure the reliability and accuracy of the experimental results, each sample underwent multiple measurements (≥3 times) to assess the nanohardness values. This systematic approach helped to evaluate the impact of irradiation on the mechanical properties, particularly the adhesion strength, of the coatings.

## 3. Results and Discussion

### 3.1. Cr_2_O_3_/Al_2_O_3_ Coating on Stainless-Steel Surface

**Figure 3:**
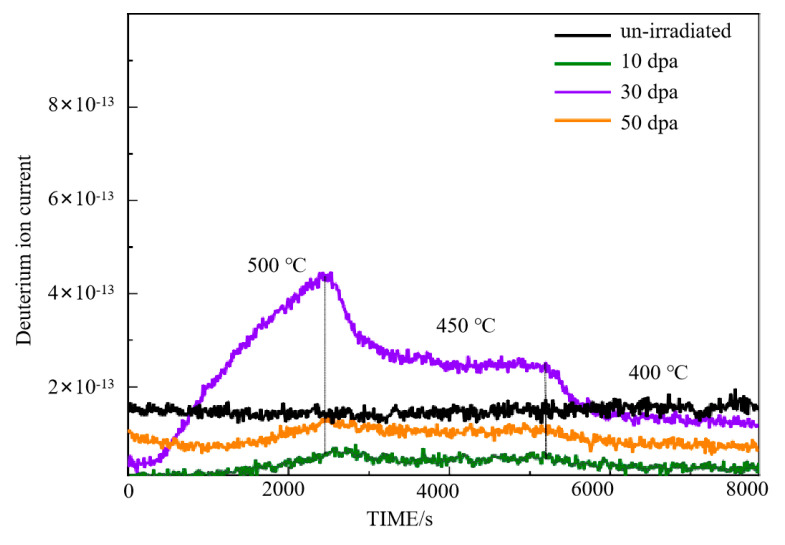
Deuterium ion current as a function of time for Cr_2_O_3_/Al_2_O_3_ coatings at different irradiation doses (10 dpa, 30 dpa, and 50 dpa) and temperatures (500 °C, 450 °C, and 400 °C; the black vertical lines indicate temperature transitions).

Figure 3 shows the deuterium permeation test curves of Cr_2_O_3_/Al_2_O_3_ composite coating after 10 dpa, 30 dpa, and 50 dpa irradiation. The test conditions are the same as those of the unirradiated samples. As seen in the figure, the deuterium permeation signal of the Cr_2_O_3_/Al_2_O_3_ composite coating increases initially and then decreases with the increase in irradiation damage. The deuterium permeation signal of the Cr_2_O_3_/Al_2_O_3_ composite coating at 10 dpa and 50 dpa of irradiation damage is lower than that of the unirradiated samples, and the change in the deuterium permeation signal is not significant as the temperature decreases. The deuterium permeation signal of the composite coating with 30 dpa of irradiation damage is significantly higher than that of the unirradiated samples at 500 °C, and decreases with the reduction in test temperature, becoming similar to the irradiated samples at 400 °C.

The permeation data presented in Figure 3 show a non-linear relationship between deuterium permeation and irradiation dose, with the highest permeation rate observed at 30 dpa, while 10 dpa and 50 dpa irradiation result in lower permeation rates than the unirradiated sample. This behavior can be explained by the complex evolution of irradiation-induced defects:

(1) At low doses (10 dpa), point defects and dislocation loops act as trapping sites for deuterium atoms, reducing the overall permeation rate. This is reflected in the microstructural observations, where grain boundary swelling and defect formation are seen, effectively hindering deuterium diffusion.
(2) At intermediate doses (30 dpa), some defects begin to recombine, and the deformation of the grain boundaries may occur, creating additional diffusion paths. This increases the permeation rate, as seen in both the permeation data and the TEM images, which show increased grain boundary deformation and more pronounced interface changes.
(3) At higher doses (50 dpa), the formation of larger voids and significant grain swelling, as observed in the SEM and TEM images, creates barriers to diffusion. These voids and other irradiation-induced defects become substantial enough to block deuterium migration, resulting in a decreased permeation rate compared to both the unirradiated and 30 dpa samples.

This non-linear behavior demonstrates the complex dynamic evolution of irradiation-induced defects and their significant impact on deuterium diffusion through the material. The SEM and TEM observations confirm that defect types, such as point defects, dislocation loops, and voids, play a crucial role in determining permeation behavior under different irradiation conditions.

**Figure 4:**
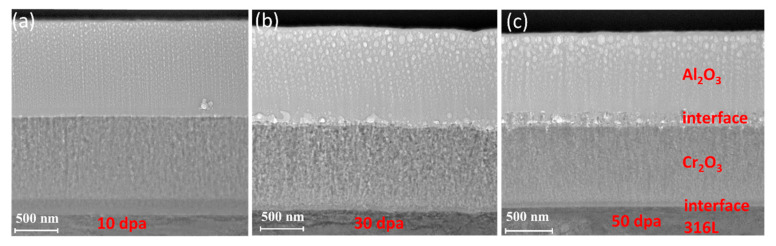
Cross-section SEM image of Cr_2_O_3_/Al_2_O_3_ composite coating: (**a**) 10 dpa, (**b**) 30 dpa, (**c**) 50 dpa.

Using FIB, the cross-section of the Cr_2_O_3_/Al_2_O_3_ composite coating was obtained, as shown in Figure 4a–c, which display the cross-sectional microstructure of the Cr_2_O_3_/Al_2_O_3_ composite coating. The Cr_2_O_3_/Al_2_O_3_ composite oxide layer exhibits good adhesion, with a uniform structural distribution within a certain depth range (~1.5 μm) on the surface layer. The composite coating is ~1.5 μm thick, with two interfacial structures: one between the substrate and Cr_2_O_3_ and the other between Cr_2_O_3_ and Al_2_O_3_. Under all three irradiation conditions (10 dpa, 30 dpa, and 50 dpa), crystal grain swelling was observed within the outer Al_2_O_3_ coating, with the grain size increasing progressively with higher irradiation doses. Additionally, void formation was observed at the Al_2_O_3_/Cr_2_O_3_ interface. The number of voids increased by more than 35.4% from 10 dpa to 50 dpa, with the average void volume increasing from 4.35 × 10^−7^ μm^3^ to 8.24 × 10^−5^ μm^3^. The growth and coalescence of these voids contributed to an increase in interfacial roughness and potential weakening of the interface between the Al_2_O_3_ and Cr_2_O_3_ layers.

To better interpret the ion irradiation results for the Cr_2_O_3_/Al_2_O_3_ multilayer coating, it is essential to consider the non-uniform distribution of energy loss and displacement damage across the depth profile, particularly at the Cr_2_O_3_/Al_2_O_3_ and substrate interfaces. The depth profile of Au ion concentration and displacement damage indicates that electronic excitations dominate near the surface, while displacement damage becomes significant in the deeper regions, especially near the interface between the two oxides and at the substrate interface. This transition in damage characteristics suggests that damage is not uniformly distributed throughout the coating and may impact the tritium permeation resistance differently at these interfaces.

At the Cr_2_O_3_/Al_2_O_3_ interface, displacement damage peaks due to the transition in material properties, leading to localized defect accumulation. This accumulation may affect the mechanical integrity and diffusion characteristics at the interface. Similarly, at the substrate interface, the differential response to displacement damage further influences the coating’s overall performance under irradiation. These interfacial effects indicate that tritium permeation resistance could vary within the coating due to non-homogeneous damage distribution.

**Figure 5:**
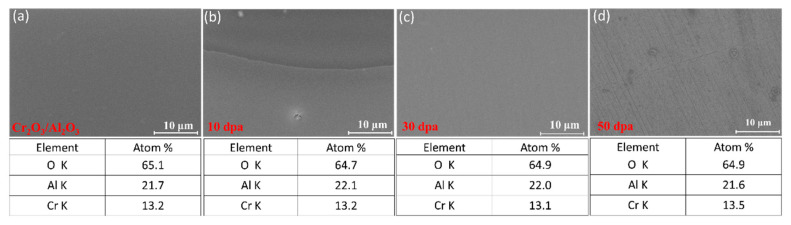
Surface SEM image of Cr_2_O_3_/Al_2_O_3_ composite coating: (**a**) unirradiated (0 dpa), (**b**) 10 dpa, (**c**) 30 dpa, (**d**) 50 dpa.

**Figure 6:**
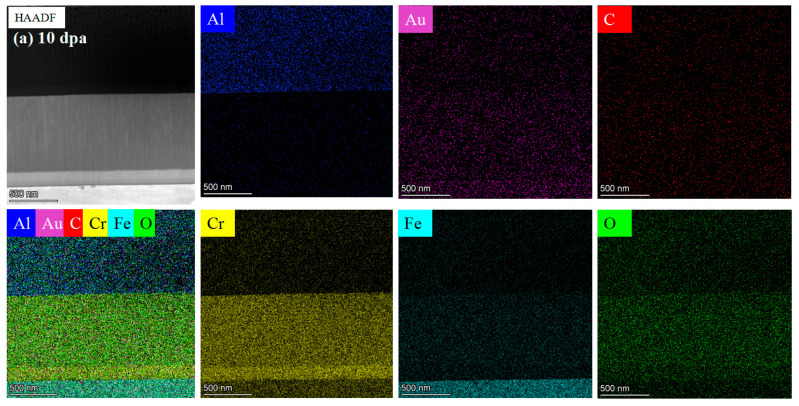

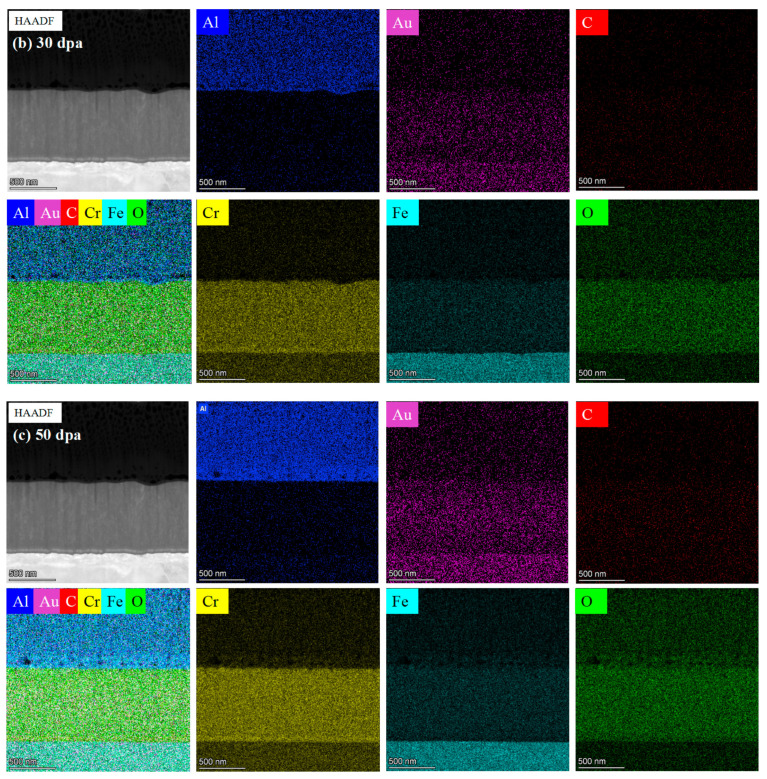
Cross-section EDS image of Cr_2_O_3_/Al_2_O_3_ composite coating: (**a**) 10 dpa, (**b**) 30 dpa, (**c**) 50 dpa.

Figure 5 and Figure 6 show the surface SEM and EDS of the Cr_2_O_3_/Al_2_O_3_ composite coating after irradiation. EDS data indicate that the bi-layer structure remains intact after irradiation, with Al, Cr, and O atoms uniformly distributed across the coating cross-section, demonstrating good radiation resistance. The atomic content of Al, Cr, and O does not significantly change with increasing irradiation dose. SEM analysis reveals that the surface of the composite coating develops a few grooves after 10 dpa irradiation and pores after 50 dpa irradiation, with increased roughness. Combined with the cross-sectional analysis, it is evident that as the irradiation dose increases, the size and density of irradiation voids increase, leading to more defects in the composite coating. At 50 dpa irradiation, significant erosion marks appear on the surface. It is also notable that, with increasing irradiation dose, the degree of swelling at the bi-layer interface of the composite coating increases. Since the composite coating interface is an effective hydrogen permeation coating, the change in deuterium permeation performance after irradiation is related to the bi-layer interface. The composite coating still demonstrates good hydrogen permeation resistance under 30 dpa irradiation, as evidenced by a comparative study with uncoated stainless steel. The permeation tests showed that, even after 30 dpa irradiation, the Cr_2_O_3_/Al_2_O_3_ bilayer coating exhibited a significantly lower hydrogen permeation rate than uncoated stainless steel under similar conditions. This indicates that the composite coating retains effective permeation resistance despite the structural changes induced by irradiation.

**Figure 7:**
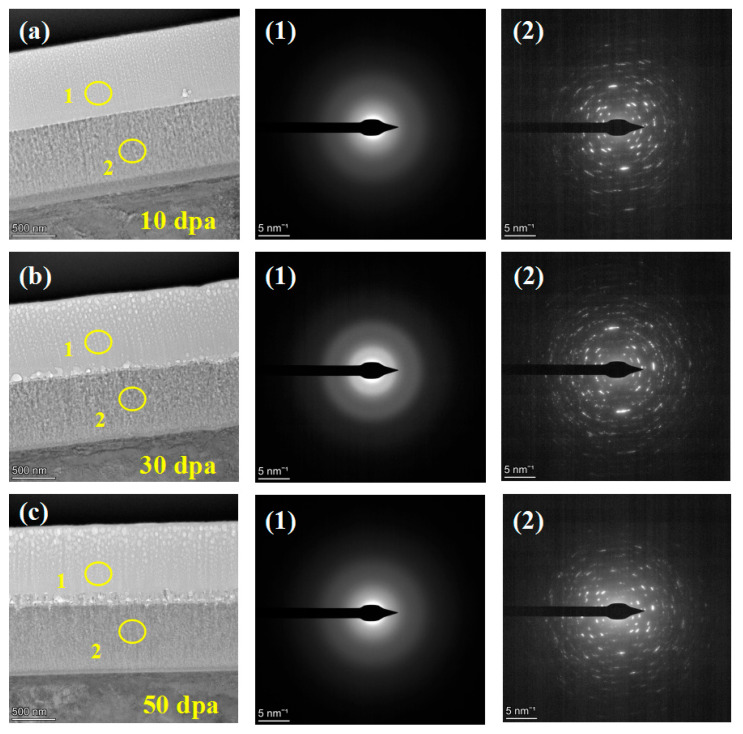
TEM cross-section image of irradiated coating and corresponding selected-area EDP: (**a**) 10 dpa, (**b**) 30 dpa and (**c**) 50 dpa.

Figure 7 shows the TEM cross-section images and SAED patterns of the irradiated coating samples. The outer layer consists of a disordered arrangement of Cr and O atoms, with the electron diffraction pattern showing severe ring dispersion, indicating a completely amorphous structure of the Cr_2_O_3_ outer layer. The inner coating consists of Al and O atoms, forming ring patterns indicative of a crystalline Al_2_O_3_ structure.

The nanoindentation tests showed that the Cr_2_O_3_/Al_2_O_3_ coating exhibited a hardness of 10.6 GPa before irradiation, which was higher than the irradiated samples, indicating better wear resistance. After irradiation at doses of 10 dpa, 30 dpa, and 50 dpa, the hardness values decreased to 7.8 GPa, 7.3 GPa, and 7.6 GPa, respectively. This decrease in hardness with increasing dose is attributed to the presence of cracks and voids observed in the TEM images. The most significant reduction at 30 dpa suggests enhanced radiation resistance from the formation of an (Al_0.9_Cr_0.1_)O_3_ phase.

### 3.2. Pre-Oxidized Zirconium Alloy

**Figure 8:**
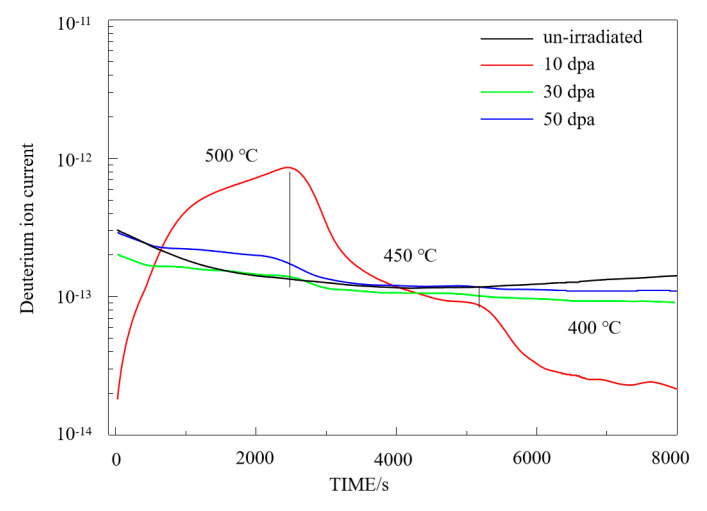
Deuterium permeation curve of irradiated pre-oxidation zirconium alloy.

Figure 8 shows the deuterium permeation test curves of a pre-oxidized zirconium alloy [20] after 10 dpa, 30 dpa, and 50 dpa irradiation. The test conditions are the same as those for the unirradiated samples. At 10 dpa, the deuterium permeation signal of the pre-oxidized zirconium alloy is significantly higher than that of the unirradiated sample at 500 °C. As the temperature decreases, the permeation signal also decreases, becoming nearly an order of magnitude lower than the unirradiated sample at 400 °C. For the 30 dpa irradiated sample, the permeation signal at 500 °C is slightly higher than that of the unirradiated sample, but as the temperature is reduced, the permeation signal becomes lower, falling below that of the unirradiated sample at 450 °C and 400 °C. For the 50 dpa irradiated sample, the permeation signal at 500 °C is higher than that of the unirradiated sample but lower than the 10 dpa irradiated sample. At 450 °C, the permeation signal of the 50 dpa sample decreases to nearly match that of the unirradiated sample, and at 400 °C, it is slightly lower than that of the unirradiated sample. At 500 °C, the deuterium permeation resistance of the samples irradiated at 10 dpa, 30 dpa, and 50 dpa decreased by approximately 1.8 times, 1.0 times, and 1.1 times, respectively, compared to the unirradiated sample.

**Figure 9:**
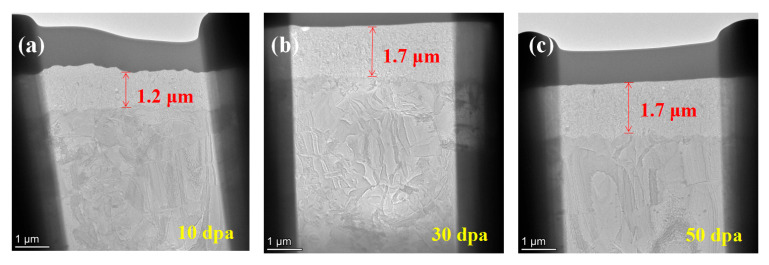
Cross-section SEM of pre-oxidation layer: (**a**) 10 dpa, (**b**) 30 dpa and (**c**) 50 dpa.

Using FIB, the cross-sectional morphological characterization of the irradiated pre-oxidation film was obtained, as shown in Figure 9. The oxide layer exhibits good adhesion to the zirconium substrate, with a uniform structural distribution in the pre-oxidation film (~1.7 μm).

**Figure 10:**
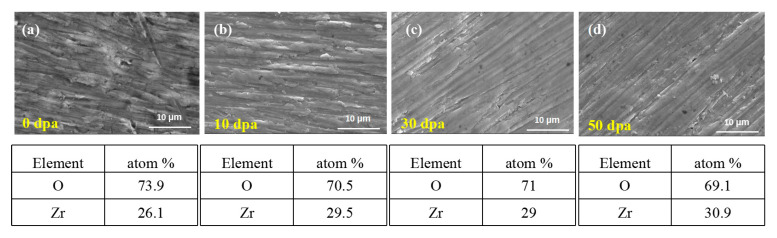
Surface SEM of pre-oxidation layer: (**a**) 0 dpa, (**b**) 10 dpa, (**c**) 30 dpa, and (**d**) 50 dpa.

**Figure 11:**
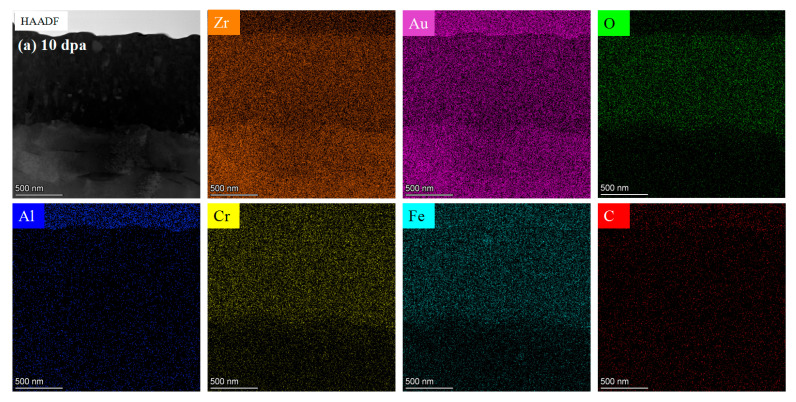

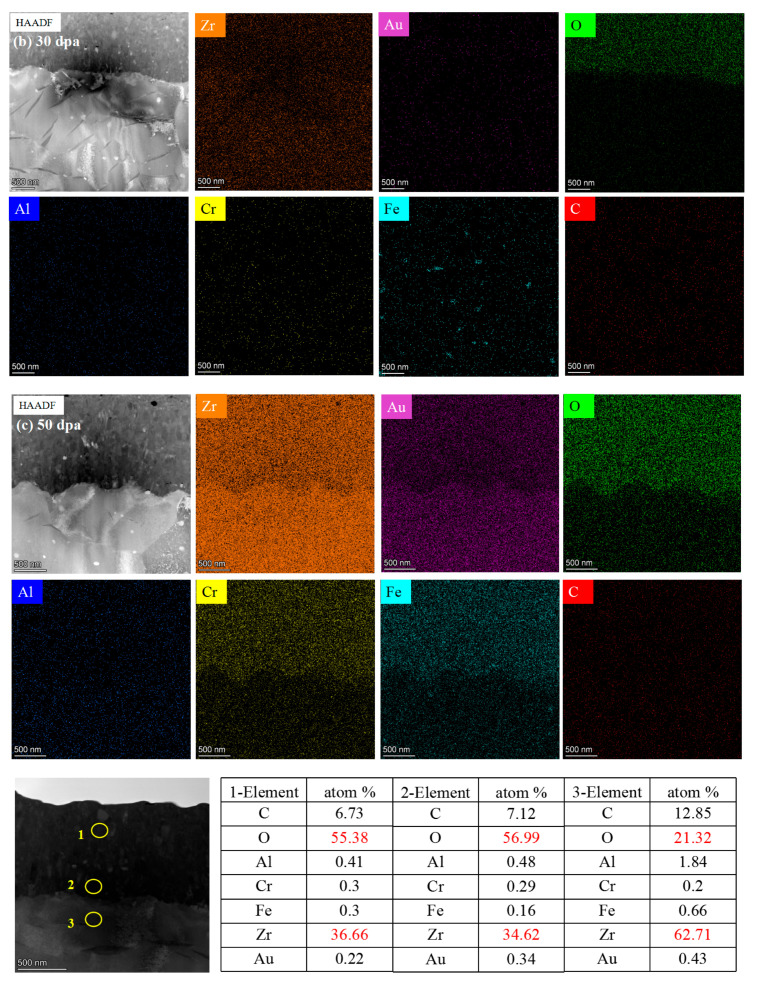
Cross-section EDS of pre-oxidation layer: (**a**) 10 dpa, (**b**) 30 dpa, and (**c**) 50 dpa.

Figure 10 and Figure 11 show the surface SEM and EDS of the pre-oxidation coating. The content of O and Zr elements on the surface of the coating does not change significantly with increasing irradiation dose, compared to the original coating surface, with a slight decrease (2~3%). This indicates that the structure of the oxidation film remains unchanged after irradiation. It is noteworthy that the coating thickness of the 10 dpa irradiated sample is only 1.2 μm, while the thickness of the 30 dpa and 50 dpa samples is nearly the same, indicating a reduction in the thickness of the oxidation film after irradiation. The 10 dpa oxidation film exhibits the largest reduction in thickness, while the 30 dpa and 50 dpa samples have higher thicknesses than the 10 dpa irradiated sample. The oxidation film formed after irradiation appears denser and smoother compared to the 10 dpa and unirradiated samples. This difference explains the different permeation curves observed for the 10 dpa, 30 dpa, and 50 dpa irradiated samples, with the 30 dpa and 50 dpa oxidation films exhibiting surface-controlled permeation and the 10 dpa sample exhibiting diffusion-controlled permeation.

The composition of the pre-oxidation film was characterized using the EDS module, as shown in Figure 11. Elemental mapping displays the distribution of O and Zr across the oxide layer cross-section under different irradiation doses, with a uniform spread of these elements indicating a dense ZrO_2_ layer. Areas 1, 2, and 3, marked in the cross-sectional view, correspond to distinct locations across the layer thickness, representing the outer, middle, and inner regions of the layer, respectively. This cross-sectional EDS image represents the sample irradiated at 10 dpa. The table in Figure 11 quantifies the relative concentrations of O and Zr in these areas, supporting the observation of compositional uniformity.

For the pre-oxidized Zr alloy, the displacement damage profile reveals that significant damage accumulates near the oxide–Zr alloy interface, where the transition in material composition creates a distinct boundary. This boundary experiences concentrated displacement damage, which may lead to structural changes and impact the tritium permeation resistance differently compared to the bulk of the oxidation film. The non-uniform distribution of damage across the depth profile, especially at the oxide–metal interface, emphasizes the importance of considering interfacial effects when evaluating tritium permeation behavior under irradiation.

**Figure 12:**
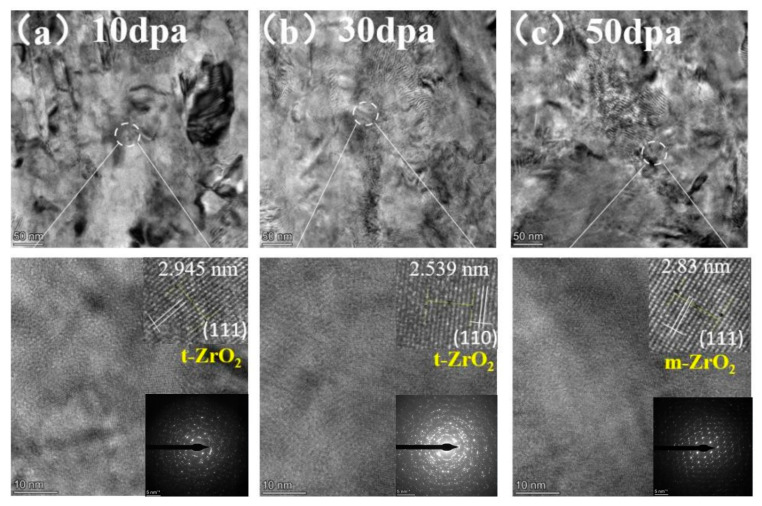
TEM cross-section image of irradiated pre-oxidation layer and corresponding selected-area EDP: (**a**) 10 dpa, (**b**) 30 dpa, and (**c**) 50 dpa.

Figure 12 shows the TEM cross-section images and SAED patterns of the irradiated coating samples. Structural changes within the pre-oxidation film were observed with increasing irradiation dose, confirming the growth of the oxidation film during irradiation.

### 3.3. Tritium Permeation Resistance Evaluation

The tritium permeation resistance of both the Cr_2_O_3_/Al_2_O_3_ composite coating and the pre-oxidized zirconium alloy was evaluated under various ion irradiation doses. The results demonstrate that both materials exhibit substantial resistance to tritium permeation, even after exposure to irradiation, as quantified by their respective PRF.

For the Cr_2_O_3_/Al_2_O_3_ coating, the PRF values were measured as 743 at 10 dpa, 193 at 30 dpa, and 579 at 50 dpa, shown in Table 3, indicating that the coating retains effective tritium permeation resistance across different irradiation doses. Notably, although the PRF decreases at 30 dpa, the coating still exhibits a PRF exceeding an order of magnitude relative to uncoated stainless steel, demonstrating its ability to inhibit tritium migration. In comparison, the pre-oxidized zirconium alloy also maintained a relatively stable tritium resistance, with PRF values of 11 at 10 dpa, 20 at 30 dpa, and 18 at 50 dpa. While the absolute PRF values for the zirconium alloy are lower than those of the Cr_2_O_3_/Al_2_O_3_ coating, they nonetheless indicate effective tritium permeation inhibition under irradiation conditions.

These results suggest that both types of coatings/pre-oxidized films continue to provide favorable tritium permeation resistance after irradiation, with PRF values above an order of magnitude. The non-linear behavior observed in PRF values across different doses may be attributed to the complex evolution of irradiation-induced defects. At lower doses, point defects and dislocation loops may capture tritium atoms, reducing permeation. At intermediate doses, some defects recombine, potentially creating diffusion pathways, while at higher doses, void formation and grain boundary swelling act as additional barriers to tritium diffusion.

In summary, the Cr_2_O_3_/Al_2_O_3_ composite coating demonstrates superior tritium permeation resistance under irradiation compared to the pre-oxidized zirconium alloy, with both materials showing substantial resilience against tritium permeation across a range of irradiation conditions.

## 4. Conclusions

The tritium permeation performance and potential mechanisms of a Cr_2_O_3_/Al_2_O_3_ composite coating on stainless steel and a pre-oxidized zirconium alloy under different irradiation doses were compared in this study. The conclusions are as follows:

1. For the Cr_2_O_3_/Al_2_O_3_ composite coating, the tritium permeation resistance decreases by approximately three times at 30 dpa but improves slightly at lower and higher irradiation doses (10 dpa and 50 dpa). The microstructure of the coating deteriorates after irradiation, with increasing defects within the layers and at the interfaces with increasing irradiation dose. These defects may increase deuterium trapping sites, slightly improving tritium permeation resistance at high irradiation doses.
2. For the pre-oxidized zirconium alloy, the tritium permeation resistance shows little change after 10 dpa, 30 dpa, and 50 dpa irradiation. The surface morphology of the oxidation film remains unchanged after irradiation, but structural transformations occur within the film with increasing irradiation dose.

Despite the observed irradiation defects in the Cr_2_O_3_/Al_2_O_3_ composite coating and the pre-oxidized zirconium alloy under high-dose irradiation environments, both types of tritium barriers demonstrated substantial tritium permeation resistance. The results indicate that, while tritium permeation resistance is retained, the non-uniform distribution of damage—particularly at the interfaces between the Cr_2_O_3_ and Al_2_O_3_ layers, as well as the oxide and Zr alloy substrate—could influence their long-term performance. Displacement damage was observed to peak at these interfaces, potentially impacting the mechanical integrity and diffusion characteristics, which should be considered in the design and application of tritium barriers.

This study provides valuable reference data for the design of tritium barriers and presents a potential candidate scheme for future engineering applications. However, for further engineering implementation, neutron irradiation tests in experimental reactors are recommended to comprehensively assess the stability and durability of these barriers, especially to verify the effects of interfacial damage under realistic reactor conditions.

## Figures and Tables

**Table 1:** Ion irradiation experimental parameters for coating samples.

Ion Type	Irradiation Temperature	Radiation Damage Dose	Particle Beam Flow Parameters
Au	450 °C	10 dpa, 30 dpa, 50 dpa	Beam type: scanning beam mode; Beam half-height width: 1 mm; Scanning area: 5 cm × 5 cm; Scanning frequency: 1 kHz; Beam current inhomogeneity: <1%; Irradiation damage dose: real-time monitoring

**Table 2:** Deuterium permeation testing conditions.

Temperature	Pressure	
500 °C	100 kPa	500 kPa
450 °C	100 kPa	500 kPa

**Table 3:** PRF results after ion irradiation tests with coatings and pre-oxidized layer at 100 kPa and 500 °C.

Sample Type	Irradiance Dose		
	10 dpa	30 dpa	50 dpa
Cr_2_O_3_/Al_2_O_3_ coating	743	193	578
Pre-oxidation layer	11	20	18

## Data Availability

The original contributions presented in the study are included in the article, further inquiries can be directed to the corresponding authors.
